# Comparison of time and cost between conventional surgical planning and virtual surgical planning in orthognathic surgery in Korea

**DOI:** 10.1186/s40902-019-0220-6

**Published:** 2019-09-12

**Authors:** Si-Yeon Park, Dae-Seok Hwang, Jae-Min Song, Uk-Kyu Kim

**Affiliations:** 1 Department of Oral and Maxillofacial surgery School of dentistry, Pusan National University, Busandaehak-ro, Mulgeum-eup, Yangsan, 50612 Republic of Korea; 2 Dental Research Institute, Pusan National University Dental Hospital, Busandaehak-ro, Mulgeum-eup, Yangsan, 50612 Republic of Korea

**Keywords:** Conventional surgical planning, Virtual surgical planning, Orthognathic surgery

## Abstract

**Background:**

The purpose of this study was to measure the time of the conventional surgical planning (CSP) and virtual surgical planning (VSP) in orthognathic surgery and to compare them in terms of cost.

**Material and method:**

This is a retrospective study of the patients who underwent orthognathic surgery at the Pusan National University Dental Hospital from December 2017 to August 2018. All the patients were analyzed through both CSP and VSP, and all the surgical stents were fabricated through manual and three-dimensional (3D) printing. The predictor variables were the planning method (CSP vs. VSP) and the surgery type (group I: Le Fort I osteotomy + bilateral sagittal split osteotomy [LFI+BSSO] or group II: only bilateral sagittal split osteotomy [BSSO]), and the outcomes were the time and cost. The results were analyzed using the paired *t* test.

**Results:**

Thirty patients (12 females, 18 males) met the inclusion criteria, and 17 patients were excluded from the study due to missing or incomplete data. There were 20 group I patients (LFI+BSSO regardless of genioplasty) and 10 group II patients (BSSO regardless of genioplasty). The average time of CSP for group I was 385 ± 7.8 min, and that for group II was 195 ± 8.33 min. The time reduction rate of VSP compared with CSP was 62.8% in group I and 41.5% in group II. On the other hand, there was no statistically significant cost reduction.

**Conclusions:**

The time investment in VSP in this study was significantly smaller than that in CSP, and the difference was greater in group I than in group II.

## Background

It is obvious that accurate and delicate patient analysis must precede successful orthognathic surgery. Patient analysis is done through thorough patient data analysis.

Conventional surgical planning (CSP) is based on the patient’s facial photographs, deformity analysis through two-dimensional (2D) cephalometric tracing, mounted cast analysis using facebow transfer, and model surgery. At first, a resident made a surgical stent based on the model surgery. At present, however, the surgeons request a laboratory to fabricate a surgical stent after sending to it the mounted cast and the surgical plan.

With the development of cone-beam computed tomography (CBCT) and of the computer-aided design and computer-aided manufacturing (CAD-CAM) technology, the virtual surgical planning (VSP) method for 3D planning and analysis has been expanding of late. VSP consists of analyzing the patient’s skeletal deformity with a 3D analysis program using CBCT, performing virtual surgery, and then fabricating a surgical stent using a 3D printing machine.

Many studies have shown that VSP has higher accuracy than 2D CSP, and the 3D analysis program that is used in it has become diversified and popularized.[1–6] It is continuously reported that VSP not only has higher accuracy, but also has a shorter time and a lower cost than CSP [7–9].

This study was conducted to determine if the time and cost difference between VSP and CSP in South Korea is the same as that reported in other countries.

## Materials and methods

### Study design and patients

This is a retrospective study of patients who underwent orthognathic surgery at OOOO University Dental Hospital from December 2017 to August 2018. Patients who (1) underwent both Le Fort I osteotomy and bilateral sagittal split osteotomy (LFI+BSSO) or only bilateral sagittal split osteotomy (BSSO) were enrolled in the study. Genioplasty was not considered. In addition, (2) preoperative preparation was performed in the oral and maxillofacial surgery (OMS) department of the authors’ hospital, and (3) surgical planning was done through both CSP and VSP. The patients with a craniofacial deformity (e.g., cleft lip and palate) and those with a previous history of head trauma or with a systemic disease were excluded from the study. The patients were divided into two groups: group I, which included patients who had undergone both LFI and BSSO regardless of genioplasty, and group II, which included patients who had undergone BSSO regardless of genioplasty.

This study was reviewed by the Institutional Review Board (IRB) of Pusan National University Dental Hospital, and was approved after thorough deliberation (PNUDH-2019-002).

### Progress workflow in CSP vs VSP

All the patients underwent radiography (panorama, lateral cephalogram, posterior-anterior cephalogram, and cone-beam computed tomography (CBCT)). Clinical photographs of them were also taken, and they were interviewed. All the cases were analyzed using both CSP and VSP.

In group I, two pairs of maxillary and mandibular impression, and facebow transfer, were needed for CSP. Impression and pouring were done by an intern, and facebow transfer was done by 2-year residents (R2). The radiographs were analyzed by R2 using the 2D analysis program V-ceph (version 6.0; Osstem, Seoul, South Korea). Then Hanau articulator mounting and simple articulator mounting were performed by an intern.

In group II, only one pair of maxillary and mandibular impression was needed for CSP, and facebow transfer was not needed. The other steps were the same as with group I.

**Fig. 1:**
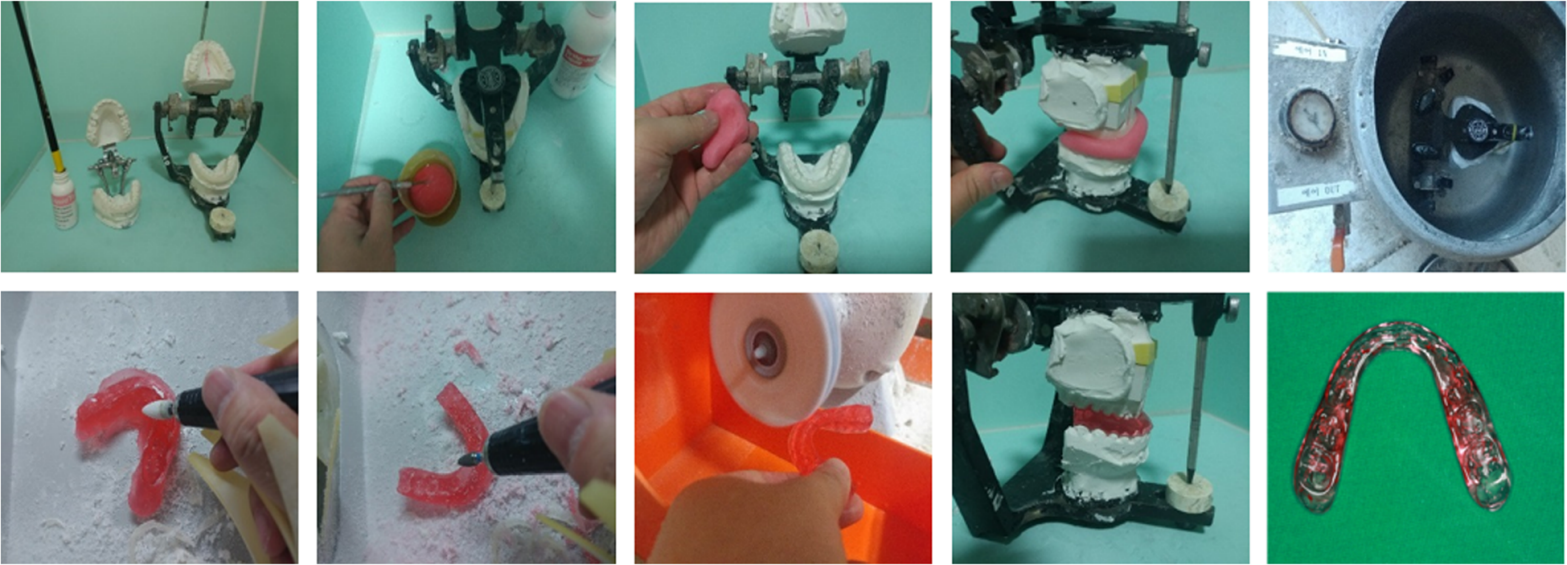
Stent fabrication progress in conventional surgical planning

The mounted casts and final occlusion were then sent to a laboratory, and the dental technician made an intermediate stent (group I only) and a final stent based on the surgical plan (Fig. 1).

**Fig. 2:**
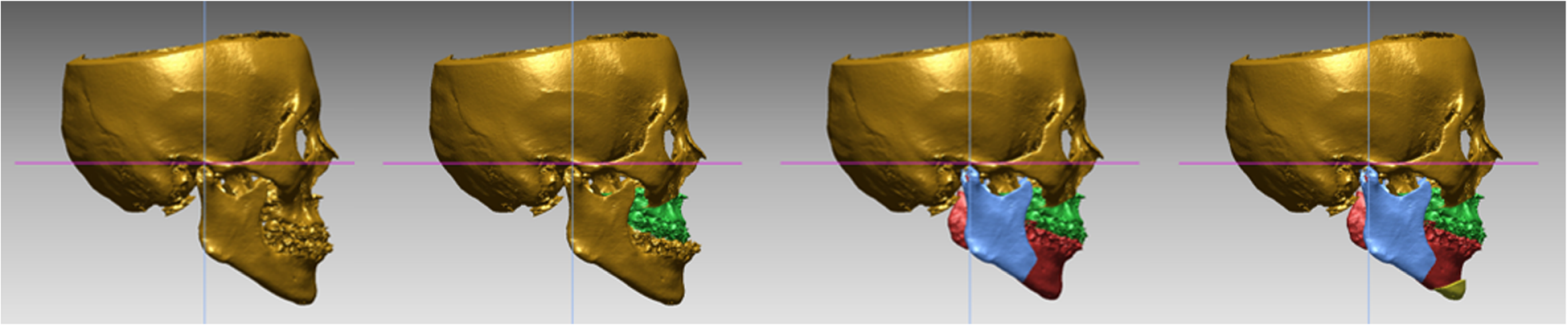
Virtual surgery in virtual surgical planning

In both groups I and II, only one pair of maxillary and mandibular impression was needed for VSP and facebow transfer was not needed. The obtained CBCT image was analyzed by an R2 using the InVivo 3D imaging software (version 6.0; Anatomage, San Jose, CA), and mounted casts and the final occlusion images were sent to a 3D printing laboratory (TRUEM Inc., Seoul, South Korea). VSP was completed with a case confirmation web meeting between the surgeon and the technician through a virtual operated model (Fig. 2). The 3D printing laboratory fabricated an intermediate stent (group I only) and a final stent using the 3D printing process, based on the surgical plan (Table 1).

**Table 1:** Progress workflow and performer for CSP and VSP

Outpatient workup	CSP		VSP		Performer
	Clinical photograph				Dental hygienist
	2-D radiography (panorama, Lat-cephalogram, PA-cephalogram) Cone-beam computed tomography (CBCT)				Technician
	Interview				Resident
	Group I	Group II	Group I	Group II	
Impression (pairs)	2	1	1		Intern
Facebow transfer	Yes	No	No		Resident
Bite registration	2	1	1		Intern
Office workup	Import Lat.-cephalogram		Import CBCT		Resident
	Trace 2-D Lat.-cephalogram		Trace 3-D CBCT		
	2-D surgical planning		3-D surgical planning		
Mounting	Hanau articulator Simple articulator	Simple articulator	Simple articulator		Intern
Laboratory	Model surgery		Virtual surgery		Resident/technician
Case confirm	No		Yes	No	
Stent Fabrication	Manual fabrication		3-D printing		

*CSP* conventional surgical planning, *VSP* virtual surgical planning, *2-D* 2-dimentional, *3-D* 3-dimentional, *Lat.-cephalogram* lateral-cephalogram, *PA-cephalogram* posterior-anterior cephalogram

### Time and costs analysis

All the steps were timed and recorded, and the times of all the steps were averaged.

The cost was analyzed by calculating the hourly rate based on the average annual salary by occupation announced by Korea Research Institute for the 2012 Vocational Education and Training Analysis of Continuing Professional Education (CPE) for Licensed National Qualifications [10]. The hourly rate (South Korean won: KRW per hour) was 42,830 KRW for the dentist, 12,390 KRW for the dental hygienist, and 14,540 KRW for the dental technician. The total cost was derived by multiplying the hourly cost by the work time depending on the occupation of the conductor in each process. The cost of stent fabrication includes the cost of software, hardware, and material used in the process at the outsourced laboratory.

### Statistical analysis

The differences between the groups were compared via a paired *t* test. *P* < 0.05 was considered to indicate statistical significance. All the data were analyzed using the SPSS software (23.0; SPSS, Chicago, IL).

## Results

A total of 47 patients underwent orthognathic surgery by one surgeon in the authors’ department during the study period. Thirty patients (12 females, 18 males) met the inclusion criteria, and 17 patients were excluded due to missing or incomplete data. There were 20 group I patients (LFI+BSSO regardless of genioplasty) and 10 group II patients (BSSO regardless of genioplasty). Two different dental hygienists, four technicians (2 radiographers, 2 dental technicians), six 2-year residents (R2), and eight interns were involved in the cases. The average times of all the steps in the workflow of CSP and VSP for group I and II are shown in Table 2. Overall, VSP takes less time than CSP. The average time of CSP in group I was 385 ± 7.8 min, and that in group II was 195 ± 8.33 min. The average time of VSP in group I was 143.2 ± 7.6 min, and that in group II was 114.1 ± 7.12 min (Table 2). When the time reduction rates were compared by category, it was found to be highest in the laboratory process. In group I, the time reduction rate was negative at office workup step, and VSP seems to have taken longer, but the difference was not statistically significant (Table 3). The average cost of CSP in group I was 805,015 KRW, and that in group II was 508,061 KRW. As for VSP, its average cost in group I was 885,905 KRW, and that in group II was 624,267 KRW (Table 4). The overall cost reduction rate was − 9.1% in group 1 and − 18.6% in group 2, and the cost reduction rates by category are shown in Table 5.

**Table 2:** Average time for each step in CSP and VSP

		CSP		VSP	
Category	Step	Group I	Group II	Group I	Group II
Outpatient workup	Clinical photograph	7.76 ± 0.3			
	2-D radiography & CBCT	5.32 ± 0.21			
	Interview	10.09 ± 1.15			
	Impression	44.62 ± 3.57	23.42 ± 3.5	22.57 ± 3.5	
	Facebow transfer	2.69 ± 0.38	–	–	–
	Bite registration	1.55 ± 0.16	0.75 ± 0.15	0.71 ± 0.15	
Subtotal		74.3 ± 4.03	48.6 ± 2.56	48.3 ± 2.61	
Office workup	Surgical planning	23.33 ± 1.89	20.15 ± 1.89	30.20 ± 1.60	25.13 ± 1.60
	Mounting (Hanau articulator)	8.23 ± 2.09	–	–	–
	Mounting (simple articulator)	3.95 ± 1.19	3.82 ± 1.15	3.73 ± 1.02	3.87 ± 1.09
Subtotal		34.4 ± 3.12	24.5 ± 2.11	35.2 ± 1.78	29.4 ± 1.81
Laboratory	Case confirm	–	–	8.93 ± 1.15	6.38 ± 1.15
	Stent fabrication	296 ± 6.7	123 ± 5.6	59.71 ± 2.21	31.24 ± 1.71
Subtotal		296 ± 6.7	123 ± 5.6	68.93 ± 3.2	36.38 ± 2.7
Total		385 ± 7.8	195 ± 8.33	143.2 ± 7.6	114.1 ± 7.12

*CSP* conventional surgical planning, *VSP* virtual surgical planning, *2-D* 2-dimentional, *3-D* 3-dimentional, *CBCT* cone-beam computed tomography

**Table 3:** Time reduction rate for each category in VSP comparing with CSP

Category	Step	Group I (%)	Group II (%)
Outpatient workup	Clinical photograph	–	
	2-D radiography and CBCT		
	Interview		
	Impression	49.0*	3.6
	Facebow transfer	100.0*	–
	Bite registration	54.2*	5.4
Subtotal		35.0*	1.6
Office workup	Surgical planning	− 1.3	− 1.25
	Mounting (Hanau articulator)	100.0*	–
	Mounting (simple articulator)	5.6	− 1.0
Subtotal		− 2.3	14.8*
Laboratory	Case confirm	− 100	− 100
	Stent fabrication	79.8*	74.6*
Subtotal		76.7*	70.4*
Total		62.8*	41.5*

*CSP* conventional surgical planning, *VSP* virtual surgical planning

*Statistically significant (*P* < 0.01)

**Table 4:** Total cost for each group in CSP and VSP

Category	Step	CSP		VSP	
		Group I	Group II	Group I	Group II
Outpatient workup	Clinical photograph	1602			
	2-D radiography and CBCT	1289			
	Interview	7203			
	Impression	31,851	16,718	16,111	
	Facebow transfer	1920	–	–	–
	Bite registration	1106	535	507	
Subtotal		44,972	27,348	26,712	
Office workup	Surgical planning	16,654	14,384	21,558	17,939
	Mounting (Hanau articulator)	5875	–	–	–
	Mounting (simple articulator)	2820	2727	2663	2763
Subtotal		25,348	17,111	24,220	20,701
Laboratory	Case confirm	–	–	6375	4554
	Stent fabrication	211,295	87,802	48,998	22,300
Stent cost		523,400	375,800	779,600	550,000
Subtotal		734,695	463,602	834,973	576,854
Total cost, KRW		805,015	508,061	885,905	624,267

*CSP* conventional surgical planning, *VSP* virtual surgical planning, *KRW* South Korea Won

**Table 5:** Cost reduction rate for each category in VSP comparing with CSP

Category	Step	Group I (%)	Group II (%)
Outpatient workup	Clinical photograph	–	
	2-D radiography and CBCT		
	Interview		
	Impression	49.6*	3.6
	Facebow transfer	100*	
	Bite registration	54.2*	5.2
Subtotal		40.6*	2.3
Office workup	Surgical planning	− 22.7*	− 20.2*
	Mounting (Hanau articulator)	100*	–
	Mounting (simple articulator)	5.6	− 0.001
Subtotal		4.5	− 17.3
Laboratory	Case confirm	− 100*	− 100*
	Stent fabrication	76.8*	74.6*
Stent cost		− 32.9*	− 31.7*
Subtotal		− 12.0	− 19.7*
Total		− 9.1	− 18.6*

*CSP* conventional surgical planning, *VSP* virtual surgical planning

*Statistically significant (*P* < 0.01)

## Discussion

**Fig. 3:**
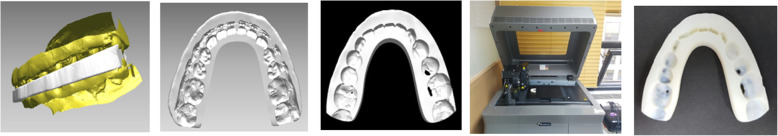
Stent fabrication progress in virtual surgical planning

CSP is often carried out manually; thus, errors and distortions often occur. For example, the impression accuracy may vary depending on the impression material mixing time, the mixing temperature, and the work time during impression, and the accuracy of the facebow transfer may vary depending on the skill of the operator and the degree of patient cooperation [11, 12]. VSP is emerging as a way of reducing errors and improving the accuracy of the surgical planning process. It is much more accurate than CSP because it involves virtual surgery and surgical stent fabrication using CBCT [5, 6, 13–16]. Due to the high accuracy of VSP, OMS surgeons use it more often than CSP when performing maxillofacial reconstruction as well as orthognathic surgery (Fig. 3). Some studies have pointed out, however, that the cost of VSP is higher than that of CSP [17–19].

This study was conducted to compare the time and cost investments in CSP and VSP in planning orthognathic surgery in South Korea. Wrozosek et al. and Resnick et al. hypothesized that VSP is more time- and cost-efficient than CSP [7, 9]. These authors aimed to categorize each step of the surgical plan between the two groups and to measure and compare the times of CSP and VSP.

In terms of the total time, that of VSP was much shorter than that of CSP in both groups in this study, and the time reduction rate was larger in group I than in group II (Table 2). This is because the processes of CSP and VSP are similar in group II, and the process of CSP in group I is largely omitted in VSP. In the office workup category in group I, the time reduction rate was negative. The difference, however, was statistically insignificant, and it can thus be concluded that CSP and VSP are similar in terms of the time to the office workup. This is similar to the results of the study of Steinhuber et al., where the time for analyzing the patients and that for planning the surgery were similar regardless of the type of program used by the OMS residents [8], as the planning is done by the patient’s characteristic and knowledge of surgeons rather than the method used.

Since the total cost of VSP was much higher than CSP in both groups, it seems likely to consider VSP was not effective in both groups (Table 4). The reason why the cost of VSP was higher than CSP was that the stent was fabricated in an outsourced laboratory instead of fabricating the stent in a dental hospital. However, when the labor cost of resident and intern was considered, the cost of VSP was much lower than CSP. Therefore, from the OMS surgeon’s point of view, when comparing all of these factors, VSP is more cost-effective than CSP.

It was found that relatively complex surgery was more time-effective than relatively simple surgery in group I; as such, it is concluded that the more complex the surgery is, the more time-effective VSP is. Otherwise, in the case of relatively simple surgery, it can be concluded that CSP is more cost-effective than VSP.

A law for the improvement of the residents’ training environment and status was recently established in South Korea. The law ensures that residents do not work for more than 80 h a week and have at least 1 day off per week. VSP does not significantly reduce the office workup time, but it saves on the resident work time by significantly reducing the laboratory work time. The transition from CSP to VSP in surgery planning can be said to be in accordance with the above trend. Many studies have shown that VSP has high accuracy, and it was also shown in this study that it is more time-effective than CSP in South Korea.

Even if VSP is more effective compared to CSP, it still has cost disadvantage due to the high cost of processing its software and hardware. However, when the number of surgeon and hospital using VSP for their surgery increases, there will be more outsourced laboratories and systems available with lower cost. Therefore, VSP will eventually be available in more effective way, and it will also increase accuracy of orthognathic surgery in South Korea.

In this study, each step of VSP and CSP was not performed in the same place. Since it was performed separately, it is possible that its accuracy and cost-effectiveness decreased when it was processed in different laboratories. As a result, if the hospital is well equipped with software and hardware, each step of VSP and CSP can be performed in the same hospital and it will increase the cost-effectiveness and accuracy of the process by reducing errors and extra charges from the outsourced laboratory.

## Conclusion

In conclusion, this study showed that VSP is more time-effective than CSP in South Korea, as is the case in other countries. With its high accuracy and time efficiency, VSP is the future for orthognathic surgery planning. As the VSP program continues to evolve, research on how to reduce the work time and cost for each step should be done.

## Data Availability

Readers interested in the data should contact the authors.

## Abbreviations

2D: Two-dimension
3D: Three-dimension
BSSO: Bilateral sagittal split osteotomy
CAD-CAM: Computer-aided design and computer-aided manufacturing
CBCT: Cone-beam computed tomography
CSP: Conventional surgical planning
LFI: Le Fort I osteotomy
OMS: Oral and maxillofacial surgery
VSP: Virtual surgical planning
